# Navigating Neurological Complications in Aesthetic Dermatology: Onset of Trigeminal Neuralgia Following Laser Hair Reduction

**DOI:** 10.7759/cureus.98112

**Published:** 2025-11-29

**Authors:** Avinash Pravin, Alex J

**Affiliations:** 1 Dermatology, Pravin Skin Clinic, Nagercoil, IND; 2 Neurology, Kanyakumari Government Medical College, Nagercoil, IND

**Keywords:** aesthetic dermatology, facial pain, iatrogenic injury, laser hair reduction, trigeminal neuralgia

## Abstract

Laser hair reduction (LHR) is a commonly performed procedure in aesthetic dermatology, but its potential to trigger neurological complications remains underrecognized. Trigeminal neuralgia is a chronic neurologic pain disorder causing sudden, intense, electric shock-like facial pain along the trigeminal nerve, which can be caused by a blood vessel compressing on the nerve or due to multiple sclerosis. This case report describes a 28-year-old woman who developed classic symptoms of trigeminal neuralgia following her third session of LHR on her upper lip. A triple-wavelength diode laser with contact cooling was used, and this procedure was initially uneventful. Within 72 hours, the patient reported unilateral radiating pain in the right maxillary region, upper teeth, and behind the eye. Thermal or mechanical stimulation of the infraorbital nerve during LHR can activate nociceptive fibers, potentially leading to the onset of neuropathic pain. The main objective of this report is to describe a rare neurological side effect of a routine dermatological procedure. It emphasizes the need for preventive strategies like bubble gum insulation and improved cooling. It also underscores the importance of multidisciplinary management and regulatory oversight, predominantly in non-medical settings where safety procedures may be inadequate. This case provides insight into raising awareness among clinicians about the potential for routine cosmetic laser procedures to cause neural injury, emphasizing the importance of anatomical precision, vigilance, and early recognition of neuropathic complications.

## Introduction

Laser hair reduction (LHR) has currently become an integral part of aesthetic dermatology. It provides long-term hair removal by means of selective photothermolysis. Anderson and Parrish developed this technique, which utilizes specific wavelengths of light to target melanin within the hair follicles [1,2]. LHR is generally considered safe and well-tolerated. But recent research reports few non-cutaneous complications like nuclear cataracts and iris atrophy in eyes, reactivation of herpes simplex infection, moderate or severe pain, and thrombophlebitis [3,4]

Neurological problems subsequent to LHR are very rare, but they are clinically significant. Trigeminal neuralgia is a type of chronic neuropathic pain characterized by paroxysmal, electric shock-like facial pain. It is usually idiopathic but can also occur secondary to neurovascular compression [5]. However, in a few susceptible individuals, certain peripheral triggers, including thermal or mechanical stimulation of sensory branches, may precipitate symptoms. Because the infraorbital nerve branches run superficially in the upper lip region, which is in close proximity to frequently treated facial areas, energy absorption or conductive heat during diode laser exposure can transiently alter nerve excitability during laser treatment procedures [6].

As per the Ministry of Health and Family Welfare judgment, dated 4th May 2023 (District Consumer Disputes Redressal Commission, Central Mumbai), the minimum qualification for conducting LHR procedure is MD, dermatology [7]. However, LHR is often carried out in non-medical settings where safety protocols might not be always followed. This creates concerns regarding the complications that may arise especially the one involving irritation of cranial nerve.In this case report we present a rare case of idiopathic trigeminal neuralgia. The objective of this case is to highlight trigeminal neuralgia as a rare yet important neurological complication following LHR and to discuss preventive and management strategies.

## Case presentation

A 28-year-old healthy female reported to the dermatology outpatient department with Fitzpatrick skin type-5 for her third LHR session focusing on her upper lip and chin. Her preceding two sessions had no reported side effects. For this procedure, triple- wavelength diode laser system, manufactured by Cocoon Medical's Primelase HR Excellence (810 nm, 940 nm, and 1060 nm) was used. The laser settings are 14 J/cm^2^ energy density, 30ms exposure time, and a frequency of 1 Hz. For minimizing epidermal damage and discomfort, contact cooling was kept active throughout the LHR session. The session was carried out smoothly with no visible side effects such as erythema, burns, or blisters. However, the patient reported a sharp, prick-like sensation localized to the philtrum instantly following a laser pulse. The sensation was temporary, so no procedural interruption was required. Therefore, the patient was discharged without any immediate signs or symptoms of discomfort.

Three days later, she returned with complaints of unilateral, radiating facial pain on the right side. The pain was a persistent, dull headache accompanied by pain radiating to the right upper teeth and gums, discomfort in the right maxillary region, and a feeling of pressure behind the right eye. The pain had gradually increased over the past 48 hours. She had no history of systemic illness, migraine, temporomandibular joint disorder, dental infections, sinusitis, or neurological conditions, and was not on any regular medication.

On examination of the cranial nerves, the olfactory nerve (CN I) was noted as normal, implying an intact sense of smell. The optic nerve (CN II) was also fully intact, as evidenced by normal visual acuity (6/6 in both eyes), normal color vision (CV), full visual fields (VF), and a healthy appearance of the retina and optic disc on fundoscopic examination. The control of eye movements, governed by the oculomotor (CN III), trochlear (CN IV), and abducens (CN VI) nerves, was confirmed as normal by full Extraocular Movements (EOM), as well as positive and symmetrical pupillary light reflexes (both direct and consensual) and an intact accommodation reflex. Sensory and motor functions of the trigeminal nerve (CN V) were normal, as shown by intact sensation over the face and positive corneal and conjunctival reflexes; the only minor finding was that the jaw jerk reflex was not elicitable, which was considered a common normal variant, especially since other motor components of CN V were intact. Moving to the facial nerve (CN VII), the symmetry of the mouth and the normal taste perception over the anterior two-thirds of the tongue demonstrated its normal function. Hearing and balance, assessed via the vestibulocochlear nerve (CN VIII), were normal, as indicated by the Rinne's test showing air conduction (AC) greater than bone conduction (BC) and a Weber's test that did not lateralize, suggesting no conductive or sensorineural hearing loss. Finally, the functions of the lower cranial nerves, including the glossopharyngeal (CN IX), vagus (CN X), accessory (CN XI), and hypoglossal (CN XII), were all documented as normal, with no signs of weakness, atrophy, or deviation of the tongue, thus completing the picture of a comprehensively normal cranial nerve assessment. Further sensory system examination revealed touch, pain, temperature, vibration, Joint position sense, and time vibration to be normal, and a negative Romberg's test.

She reported that initial treatment with anti-inflammatory medications did not control pain. The visual analogue scale (VAS) score was assessed to be 6. An MRI of the brain was performed, and it was found to be normal. Because of the nature and location of the pain, a neurological cause was suspected, and the patient was referred to a neurologist. Following a comprehensive assessment, dental pathology and sinusitis were ruled out. Given the timing of the laser procedure, location of the infraorbital nerve, and absence of other identifiable causes, a probable diagnosis of laser-induced trigeminal neuralgia was made. Since the patient has normal sensory functions of CN V distribution and no motor weakness of muscles innervated by the CN V nerve, it helped us to distinguish trigeminal neuropathy from neuralgia. In trigeminal neuropathy, there is sensory involvement along with the trigeminal nerve distribution, and motor involvement is also affected. The clinical presentation met the criteria for idiopathic trigeminal neuralgia according to the International Classification of Headache Disorders, Third Edition (ICHD-3) (IHS13.1.1.3).

The choice of therapy is influenced by important safety considerations. Carbamazepine, the standard first-line treatment, is associated with a higher risk of Stevens-Johnson Syndrome (SJS) in Asian populations. Current recommendations advise genetic testing for the HLA-B1502 allele prior to initiating carbamazepine, given its strong association with SJS. To prevent this potentially life-threatening complication, amitriptyline was initiated with a two-week course, while simultaneously suggesting genetic testing to guide escalation if adequate clinical response was not achieved. This approach reflects a context-specific adaptation of management, balancing efficacy with patient safety. This completely resolved the symptoms. A VAS score of 0 and no recurrence were observed during the six-month follow-up.

## Discussion

Trigeminal neuralgia, also known as Tic Douloureux, is a chronic neuropathic pain disorder characterized by brief, electric shock-like or stabbing sensations affecting areas innervated by the trigeminal nerve [8]. It predominantly affects females, with a male:female ratio ranging from 1:1.13 to 1:3. They are classified into three types: idiopathic, classical, and secondary. In general, their cause is idiopathic, with 11% of cases showing normal neuroimaging findings [9,10]. This is usually sensitive to peripheral triggers. Pain can be provoked by minimal stimuli such as touch, temperature changes, or movement. Dermatological procedures are rarely involved but should not be excluded as probable triggers. The underlying pathophysiology involves the activation of A-delta and C fibers due to any external stimuli/triggers. Pain most often spreads through maxillary or mandibular branches, although any division or combination may be affected [11,12].

Indian skin ranges from type IV to VI on the Fitzpatrick skin type scale and is more pigmented owing to the presence of greater amounts of epidermal melanin and larger melanosomes. This makes it more vulnerable to post-inflammatory hyperpigmentation following laser treatment. The complications of laser hair reduction procedures may be classified as immediate (up to seven days), transient (one to six weeks), and persistent (after six weeks). Immediate compications include crusting, superficial thrombophlebitis, cold urticaria, palpable purpura, transient complications include acneform eruptions, folliculitis, reticulate erythema; and persistent complications include paradoxical hypertrichiosis, undesired styling of hairline, ocular compications and leukotrichia [13].

Laser irradiation can induce significant nerve injury primarily through vascular damage rather than direct axonal destruction. Diode laser exposure causes endothelial disruption, thrombosis, and reduced epineurial blood flow, leading to ischemia of underlying nerve fibers. These changes result in demyelination, axonal degeneration, and loss of both sensory and motor function, as evidenced by reduced compound muscle and sensory nerve action potentials. Functionally, affected animals exhibit neuropathic pain behaviors, including thermal hyperalgesia and mechanical allodynia, along with partial denervation of muscles and skin. Thus, laser-induced neuropathy arises mainly from secondary ischemic mechanisms following vascular injury rather than from direct thermal effects on axons [14].

According to ICHD-3 [15], idiopathic trigeminal neuralgia is diagnosed based on recurrent paroxysms of unilateral facial pain and continuous or near-continuous pain between attacks within the trigeminal distribution. Management typically begins with pharmacologic therapy. Carbamazepine remains the first-line of drug approved by the FDA. Other effective options include phenytoin, lamotrigine, amitriptyline, baclofen, gabapentin, and pregabalin [16]. Botulinum toxin A has emerged as a promising adjunctive therapy, though current evidence remains limited [17]. Surgical interventions such as microvascular decompression, gamma knife radiosurgery, glycerol rhizotomy, radiofrequency thermocoagulation, and percutaneous balloon compression are reserved for refractory cases or those with identifiable structural causes.

Both education and training are needed to prepare healthcare professionals for the safe and effective use of lasers in surgery. Not all laser safety requirements apply to every laser. Each wavelength, system, delivery device, and application must be assessed for applicable hazards, since they are all different, and will require different management and procedures. Implementation and Monitoring of Control Measures as prescribed by facility policy, and are based on the hazards identified during risk assessment [18]. Avoid use in patients with recent sun exposure, avoid application of excessive energy densities and/or pulse stacking, lower cutoff filters should be used for patients with fair skin phototypes; longer pulse durations can improve treatment efficacy and decrease risk, reserve for use in lower extremities (small-to medium-sized veins); avoid pulse stacking for nasal alae treatment [19].

**Figure 1:**
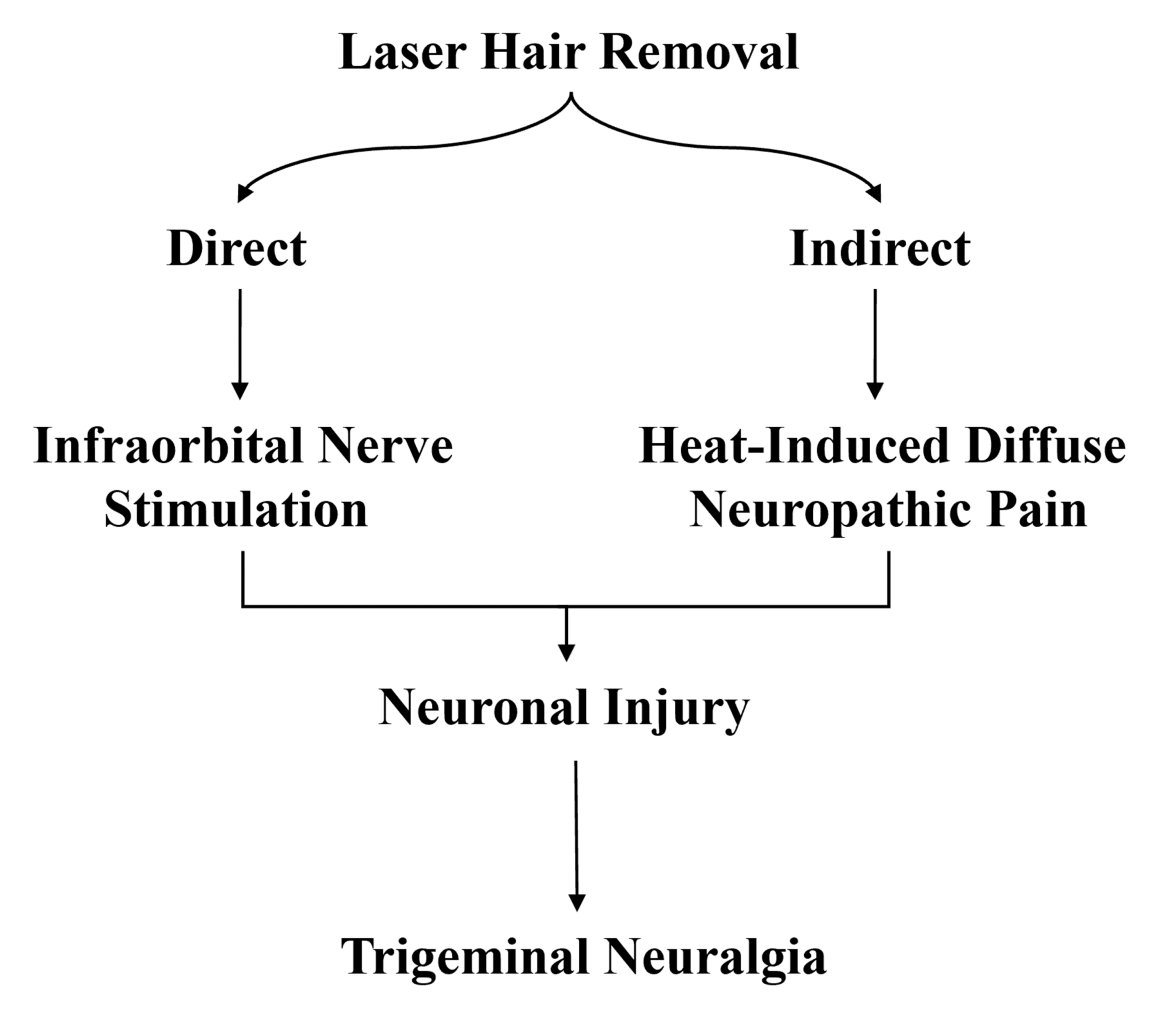
Pathways linking laser hair removal to trigeminal neuralgia Image conceptualised and created by the author.

Trigeminal neuralgia must be differentiated from autonomic cephalgias and short-lasting unilateral neuralgiform headache attacks with cranial autonomic symptoms (Short-lasting Unilateral Neuralgiform headache attacks with Conjunctival injection and Tearing/Short-lasting Unilateral Neuralgiform headache attacks with Cranial autonomic symptom (SUNCT/SUNA)). A distinguishing feature of trigeminal neuralgia is the presence of a refractory period after an attack, during which further pain cannot be provoked. But this phenomenon does not occur in autonomic cephalgias [20]. For the effectiveness of treatment and its outcomes, a multidisciplinary approach involving dermatologists, neurologists, and pain specialists is essential. The pathophysiology linking LHR to trigeminal neuralgia likely involves direct or indirect stimulation of the infraorbital nerve (Figure 1).

Furthermore, due to the proximity of sensitive oral structures and innervations, the patient may experience discomfort, particularly in the upper lip region. A similar type of persistent neuropathic facial pain following intense pulsed light hair removal was reported by Gay-Escoda et al. [21]. Traditional approaches like silicone gel sheets or gauze-covered wooden spatulas have limitations in practicality and patient comfort [22,23]. The use of bubble gum is considered an insulating barrier to lessen heat transfer and nerve irritation during upper lip treatments, as reported by Gupta et al. [24]. Other procedural techniques, such as adequate insulation, cooling, and patient screening, should also be considered.

## Conclusions

LHR is considered a safe, medical-grade procedure, but it can also trigger rare neurological complications like trigeminal neuralgia. These risks can be minimized using preventive measures like topical anaesthesia, active cooling, and bubble gum insulation. Thus, dermatologists need to exercise caution during LHR procedures, particularly in sensitive facial areas. Furthermore, in India, widespread misuse of laser devices in salons, often without adherence to safety protocols, is a significant concern. A dedicated regulatory body is urgently needed to enforce standards and ensure patient safety.
